# Changes in 1‐year relative survival of patients with cancer during the COVID‐19 pandemic in Denmark, Finland, Iceland, Norway, and Sweden: A population‐based cohort study

**DOI:** 10.1002/ijc.70336

**Published:** 2026-01-21

**Authors:** Fernando Gonzalez Yli‐Mäyry, Tomas Tanskanen, Karri Seppä, Anna L. V. Johansson, Charlotte Wessel Skovlund, Lina Steinrud Mørch, Søren Friis, Simon Mathis Kønig, Tom Børge Johannesen, Tor Åge Myklebust, Sasha Pejicic, David Pettersson, Eva María Guðmundsdóttir, Sirpa Heinävaara, Nea Malila, Joonas Miettinen, Johan Ahlgren, Giske Ursin, Janne Pitkäniemi

**Affiliations:** ^1^ Finnish Cancer Registry Cancer Society of Finland Helsinki Finland; ^2^ Cancer Registry of Norway Norwegian Institute of Public Health Oslo Norway; ^3^ Department of Medical Epidemiology and Biostatistics Karolinska Institutet Stockholm Sweden; ^4^ Danish Cancer Institute Danish Cancer Society Copenhagen Denmark; ^5^ Department of Research and Innovation Møre and Romsdal Hospital Trust Ålesund Norway; ^6^ National Board of Health and Welfare Stockholm Sweden; ^7^ Cancer Registry of Iceland Icelandic Cancer Society Reykjavik Iceland; ^8^ Department of Public Health, Faculty of Medicine University of Helsinki Helsinki Finland; ^9^ Regional Cancer Center Central Sweden Uppsala Sweden; ^10^ Institute of Basic Medical Sciences University of Oslo Oslo Norway; ^11^ Department of Preventive Medicine University of Southern California Los Angeles California USA; ^12^ Health Sciences Unit, Faculty of Social Sciences Tampere University Tampere Finland

**Keywords:** COVID‐19, excess mortality, Nordic countries, relative survival, SARS‐CoV‐2

## Abstract

During the first year of the COVID‐19 pandemic, reported cancer cases declined in the Nordic countries, potentially reflecting delays in cancer diagnosis. We compared 1‐year relative survival (RS) and excess mortality of patients diagnosed with cancer in the Nordic countries in March–December 2020 with that expected based on patients diagnosed in 2011–2019. We used flexible parametric RS models, defining excess mortality as the difference in total mortality between patients with cancer and the national population without cancer. We report the ratio between the observed and expected excess mortality (EMR) and the difference in 1‐year RS in percentage points (pp) by country, age, sex, and cancer site. Excess mortality of patients diagnosed during the pandemic was increased in all Nordic countries except Iceland. Swedish men had the highest EMR of 1.12 (95% CI 1.06, 1.17), corresponding to a 1.4 pp reduction in 1‐year RS (87.1%–85.8%). In women, the highest EMR was 1.10 (95% CI 1.03, 1.18) in Norway, corresponding to a 1‐year RS decrease of 1.2 pp (86.6%–85.5%). The largest site‐specific decreases in 1‐year RS were observed for liver cancer in Finnish and Swedish men, with decreases of 10.2 pp (45.3%–35.1%) and 7.2 pp (55.7%–48.5%), respectively. We found reduced 1‐year RS among Nordic patients diagnosed with cancer during the COVID‐19 pandemic in 2020, especially in older patients and those with aggressive cancers. These reductions coincided with restrictions and potential delays in seeking healthcare.

## Graphical abstract

What's new?

In the first year of the COVID‐19 pandemic, cancer reporting in the Nordic countries declined, presumably owing to delays in cancer diagnosis. How the pandemic impacted cancer survival in these countries, however, remains unclear. Using data from Nordic cancer registries that together include more than 27 million people, the authors of the present study examined 1‐year relative survival and mortality among patients diagnosed between March and December 2020. Evidence shows that survival at 1 year was reduced and mortality increased for patients diagnosed during this period. These effects were most pronounced among older patients and those with more aggressive cancers.

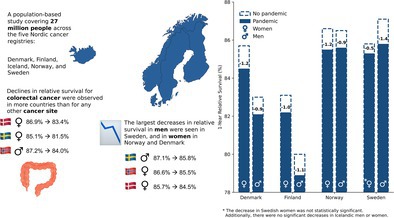

**Abbreviations**
CI: confidence interval
COVID‐19: coronavirus disease 2019
EMR: excess mortality ratio
pp: percentage point
RS: relative survival
SARS‐CoV‐2: severe acute respiratory syndrome coronavirus 2

## 1 INTRODUCTION

During the COVID‐19 pandemic, the Nordic countries (Denmark, Finland, Iceland, Norway, and Sweden) based their public health recommendations and restrictions on a foundation of solidarity and trust in institutions and fellow citizens.^1^ The pandemic caused delays in seeking healthcare, reduced diagnostic activity, and lowered cancer screening attendance, all of which may have contributed to delays in cancer diagnosis.^2^, ^3^

The COVID‐19 pandemic led to a notable increase in all‐cause mortality compared to pre‐pandemic patterns, with differences observed between the Nordic countries, as reported by the Human Mortality Database.^4^ Cumulative excess deaths since January 1, 2020, rose in Sweden early in the pandemic, whereas other Nordic countries exhibited marginal fluctuations above or below predicted mortality rates lasting until the end of 2021. By the end of 2021 excess deaths had cumulated in all Nordic countries. By the end of 2023, the highest cumulative excess deaths were reported in Finland and Iceland at 7% and the lowest in Denmark at 3%.

One‐year relative cancer survival has increased over time in the Nordic countries according to the NORDCAN database.^5^ According to the database the highest 1‐year relative survival (RS) rates for all cancer sites combined in 2012–2016 were observed in Sweden with 87.0% in men and 85.6% in women. The lowest 1‐year RS rates were seen in Finland with 80.9% in men and in Iceland with 82.0% in women. Comparing the 1‐year RS rates of men diagnosed with cancer in 2012–2016 to those diagnosed in 2007–2011, survival increased in all Nordic countries, with the increase ranging from 0.5 pp in Iceland to 4.5 pp in Denmark. In women, 1‐year RS increased similarly, ranging from 0.9 pp in Iceland to 4.7 pp in Denmark.

We have previously reported substantial and consistent declines in new cancer diagnoses in Denmark, Finland, Norway, and Sweden during the first year of the pandemic, particularly in March–June 2020, with Sweden experiencing the most pronounced declines.^2^ Although these findings suggest that the pandemic caused delays in cancer diagnosis, data on the consequences of such delays are scarce.^6^ Population‐based studies from Denmark, Iceland, and Norway indicate that patients with cancer were more likely to test positive for SARS‐CoV‐2 during the first pandemic wave and that the relative risks of severe COVID‐19 outcomes remained elevated throughout 2020.^7^

Several studies have examined cancer survival during the COVID‐19 pandemic, but results have been inconclusive, and few large population‐based studies have been published. In a study from the Belgian Cancer Registry, 1‐year RS was lower than predicted in patients diagnosed with lung and colorectal cancer in 2020, but no significant difference was observed for most cancer sites.^8^ In a study from the Alberta Cancer Registry, 1‐year overall survival was lower in patients diagnosed with colorectal cancer, non‐Hodgkin lymphoma, and uterine cancer in 2020 than in 2018, but no other major cancer sites showed significant decreases.^9^ In a study from the Northern Ireland Cancer Registry, 1‐year RS for all cancer sites was lower in patients diagnosed in April–December 2020 than in the corresponding period in 2018–2019.^10^ In a study of primary care records from the United Kingdom, 1‐year overall survival decreased for most cancer sites in patients diagnosed in 2020–2022 compared to 2015–2019.^11^

Using population‐based data from the five Nordic cancer registries, this study aims to examine the 1‐year RS and excess mortality of patients diagnosed with cancer during the COVID‐19 pandemic and to assess potential differences between the Nordic countries. The RS of patients diagnosed with cancer in the pandemic period, March–December 2020, is compared with the expected RS in the absence of the pandemic.

## 2 MATERIALS AND METHODS

The NORDCAN database provides comparable data on cancer incidence and mortality in the Nordic countries.^12^ We collected and harmonized cancer data from the national cancer registries of Denmark, Finland, Iceland, Norway, and Sweden according to the NORDCAN data call for incidence. The sources of cancer data have been described previously.^13^ Official death statistics were collected for each country participating in the study. Rates presented in Table 1 were age‐standardized using the 2013 European Standard Population to enable comparisons between countries.^14^

**TABLE 1:** Annual age‐standardized rates (per 100,000 person‐years) of cancer mortality, all‐cause mortality, and cancer incidence in men and women in the Nordic countries between 2011 and 2021.

		Denmark			Finland			Iceland			Norway			Sweden		
		Cancer mortality	All‐cause mortality	Cancer incidence	Cancer mortality	All‐cause mortality	Cancer incidence	Cancer mortality	All‐cause mortality	Cancer incidence	Cancer mortality	All‐cause mortality	Cancer incidence	Cancer mortality	All‐cause mortality	Cancer incidence
Women	2011	260	959	656	187	818	487	328	884	603	200	835	582	180	829	491
	2012–2018^a^	−4.9	−11	−1.7	−1.6	−7.4	3.4	−1.1	−12	−5.6	−0.3	−9.1	6	−1.4	−6.1	1.4
	2019	227	846	652	177	739	518	321	778	564	191	754	620	169	745	508
	2020	220	834	623	175	737	490	308	774	571	186	739	603	162	795	474
	2021	214	860	634	171	751	504	313	786	586	190	756	628	159	739	498
Men	2011	375	1364	803	299	1337	660	498	1158	683	320	1233	829	258	1183	641
	2012–2018^a^	−7.7	−15.7	−3.9	−3.9	−21.1	−0.6	−9.9	−19.7	−9.9	−5.4	−25.4	−4.4	−3.9	−14.3	0.9
	2019	324	1198	772	276	1154	654	435	1011	624	275	1032	789	227	1029	648
	2020	315	1191	743	267	1151	629	434	986	621	273	1009	781	219	1125	577
	2021	308	1212	763	269	1176	634	427	1006	637	262	1004	776	204	1035	603

*Note*: Rates are standardized to the European standard population 2013.

^a^ Average of the differences between consecutive calendar years in the numbers of cancer deaths, all deaths, and incident cancers.

Cancer entities were defined according to the NORDCAN cancer dictionary.^5^ Non‐melanoma skin cancers were excluded from all analyses. We focused on seven cancer sites selected for their high incidence or mortality (breast, prostate, colorectal, lung, pancreatic, hematopoietic, and liver). Hematopoietic cancers were combined to account for variations in registration practices across countries. Tumor stage was not used due to incomplete recording across the Nordic cancer registries.^15^ Patients were followed from the date of cancer diagnosis until death, emigration, or December 31, 2021, whichever came first. Virtually all patients had potentially complete 1‐year follow‐up. The World Health Organization characterized the COVID‐19 outbreak as a pandemic on March 11, 2020, and therefore, the pandemic period was defined as March–December 2020.

We studied relative survival and excess mortality because both measure cancer‐specific mortality, avoid the need for cause‐of‐death information, and are commonly used in population‐based cancer studies. Excess mortality is defined as the difference in the total mortality of patients and the mortality that would be expected in the absence of cancer.^16^ The expected mortality was estimated from the national population mortality rates by excluding patients with cancer and was stratified by country, sex, 5‐year age group (0–4, 5–9, …, 85–89, and ≥90 years), and calendar month. Studying relative survival and excess mortality allows us to isolate the mortality component attributable to cancer, independent of shifts in background mortality.

We estimated the pandemic effect as the difference between observed and expected 1‐year RS in the pandemic period. We censored the follow‐up of patients diagnosed in 2019 at the end of February 2020 to ensure no overlap with the pandemic period and to avoid the potential influence of care received during the early stages of the pandemic. Patients diagnosed in January or February in any of the years 2011–2020 were excluded from survival analysis because the aim was to study patients diagnosed with cancer in March–December 2020.

One‐year RS was estimated using flexible parametric RS models.^17^ Each model included a linear trend term for calendar year at diagnosis (2011–2020) and an indicator variable for diagnosis during the pandemic period. This model specification corresponds to an interrupted time series analysis that accounts for underlying survival trends expected to have occurred in the absence of the pandemic.^18^, ^19^ RS estimation permitted the effect of the pandemic period to vary over follow‐up time using a restricted cubic spline with 1 degree of freedom, whereas excess mortality ratios (EMRs) were estimated under the proportional hazards assumption. If the effect of the pandemic changed during the one‐year follow‐up, the estimate of relative survival could be considered more appropriate than that of EMR. The baseline hazard functions were allowed to vary by age at diagnosis (0–44, 45–54, 55–64, 65–74, 75–84, and ≥85 years) and were modelled using restricted cubic splines with 3 degrees of freedom on the log cumulative excess hazard. For prostate cancer, ages 0–64 years were combined into a single age group to improve model convergence. The RS models were fitted separately by country, sex, and either age at diagnosis (0–54, 55–74, ≥75 years, and all ages) or cancer site. RS estimates were standardized to the sex‐, site‐, and country‐specific age distributions of patients diagnosed in the pandemic period. We also estimated the number of excess deaths by applying the difference between the observed and expected 1‐year RS to the number of patients diagnosed in 2020. Site‐specific results are not reported for Iceland due to a low number of cancers. Including Iceland in these analyses could have led to unstable estimates and potentially misleading conclusions.

Statistical analyses were performed using R software (R Foundation for Statistical Computing, Vienna, Austria) with the packages Epi 2.46, popEpi 0.4.9, and rstpm2 1.5.5. To harmonize data analysis, a common analysis code was used in all participant countries.^20^, ^21^, ^22^

## 3 RESULTS

The age‐standardized rates for cancer mortality, all‐cause mortality, and cancer incidence in Denmark, Finland, Iceland, Norway, and Sweden are presented in Table 1. Between 2011 and 2018, all five Nordic countries experienced steady declines in cancer mortality and even larger drops in all‐cause mortality, while cancer incidence remained broadly stable. In 2020, the first pandemic year, cancer incidence fell in Denmark, Finland, Norway, and Sweden. In Iceland, incidence rose in women but fell in men. Cancer mortality continued its gradual decline, and all‐cause mortality fell further in all countries except Sweden, where it rose in women. In 2021, all‐cause mortality rose in most countries; incidence showed a general rebound toward pre‐pandemic levels while cancer mortality continued its downward trend. The absolute numbers of incident cancers, cancer deaths, and all deaths in the five Nordic countries are shown in Table S1, Supporting Information. In 2019, 167,483 incident cancers were recorded in all Nordic cancer registries combined. During the pandemic, this figure decreased to 161,679 in 2020 and increased to 168,484 in 2021. Regarding cancer deaths, 59,328 were recorded in 2019, whereas during the pandemic, 59,128 were recorded in 2020 and 58,797 in 2021.

**FIGURE 1:**
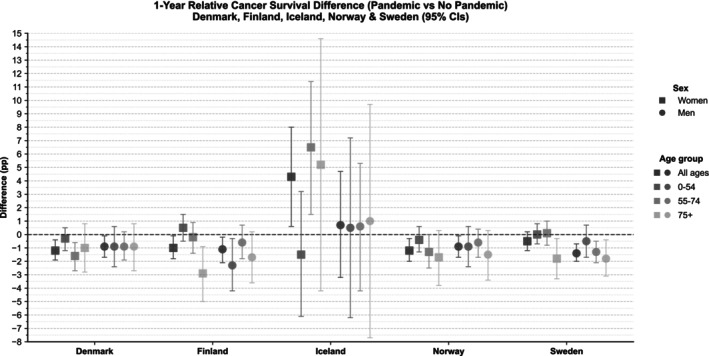
Difference in 1‐year relative survival (percentage points, pp) between patients with cancer diagnosed during the pandemic (Pandemic) and expected without the pandemic (No pandemic) for Denmark, Finland, Iceland, Norway, and Sweden, with 95% confidence intervals.

A significant reduction in 1‐year RS was seen in men in all Nordic countries except Iceland, and in women in all countries except Sweden and Iceland, with reductions ranging from 0.9 to 1.4 pp (Figure 1 and Table 2). Notably, 1‐year RS decreased by approximately 2–3 pp compared to the expected value in Finnish women aged 75 years and older, and Swedish men and women aged 75 years and older.

**TABLE 2:** One‐year relative survival (%) of patients diagnosed with cancer during the pandemic (Pandemic), the expected survival without the pandemic (No pandemic), and their difference (Difference) in percentage points (pp) with 95% confidence intervals in Denmark, Finland, Iceland, Norway, and Sweden.

		Denmark			Finland			Iceland			Norway			Sweden		
		Pandemic	No pandemic	Difference	Pandemic	No pandemic	Difference	Pandemic	No pandemic	Difference	Pandemic	No pandemic	Difference	Pandemic	No pandemic	Difference
Women	All ages	84.5 (83.9, 85.2)	85.7 (85.3, 86.1)	−1.2 (−1.9, −0.4)	82.2 (81.5, 82.9)	83.1 (82.6, 83.7)	−1.0 (−1.8, −0.1)	86.0 (83.2, 88.8)	81.7 (79.3, 84.2)	4.3 (0.6, 8.0)	85.5 (84.8, 86.2)	86.6 (86.2, 87.1)	−1.2 (−2.0, −0.3)	85.3 (84.8, 85.9)	85.8 (85.4, 86.2)	−0.5 (−1.2, 0.2)
	0–54 years	95.8 (95.0, 96.5)	96.1 (95.6, 96.6)	−0.3 (−1.2, 0.5)	96.3 (95.5, 97.2)	95.9 (95.2, 96.5)	0.5 (−0.5, 1.5)	94.2 (90.4, 97.9)	95.6 (92.8, 98.4)	−1.5 (−6.1, 3.2)	95.9 (95.1, 96.7)	96.3 (95.7, 96.8)	−0.4 (−1.3, 0.6)	96.3 (95.7, 96.9)	96.3 (95.9, 96.7)	0.0 (−0.7, 0.8)
	55–74 years	85.2 (84.3, 86.0)	86.8 (86.2, 87.4)	−1.6 (−2.7, −0.6)	86.2 (85.3, 87.1)	86.4 (85.7, 87.1)	−0.2 (−1.4, 0.9)	90.2 (86.7, 93.7)	83.7 (80.2, 87.2)	6.5 (1.5, 11.4)	86.7 (85.7, 87.7)	88.0 (87.3, 88.6)	−1.3 (−2.5, 0.0)	87.1 (86.3, 87.8)	87.0 (86.5, 87.5)	0.1 (−0.8, 1.0)
	75+ years	75.0 (73.5, 76.5)	76.0 (75.0, 77.1)	−1.0 (−2.8, 0.8)	66.9 (65.2, 68.6)	69.9 (68.7, 71.0)	−2.9 (−5.0, −0.9)	71.5 (64.2, 78.8)	66.3 (60.4, 72.2)	5.2 (−4.2, 14.6)	75.1 (73.5, 76.8)	76.9 (75.7, 78.0)	−1.7 (−3.8, 0.3)	75.1 (73.8, 76.3)	76.8 (76.0, 77.7)	−1.8 (−3.3, −0.3)
Men	All ages	82.1 (81.5, 82.8)	83.0 (82.6, 83.5)	−0.9 (−1.7, −0.1)	78.9 (78.1, 79.6)	80.0 (79.4, 80.5)	−1.1 (−2.1, −0.2)	83.3 (80.2, 86.3)	82.5 (80.1, 84.9)	0.7 (−3.2, 4.7)	85.6 (85.0, 86.3)	86.5 (86.0, 87.0)	−0.9 (−1.7, −0.1)	85.8 (85.3, 86.3)	87.1 (86.8, 87.5)	−1.4 (−2.0, −0.7)
	0–54 years	92.1 (90.9, 93.4)	93.0 (92.2, 93.8)	−0.9 (−2.4, 0.6)	89.8 (88.1, 91.4)	92.1 (91.0, 93.1)	−2.3 (−4.2, −0.3)	93.6 (88.0, 99.1)	93.1 (89.4, 96.8)	0.5 (−6.2, 7.2)	92.6 (91.4, 93.9)	93.6 (92.7, 94.4)	−0.9 (−2.4, 0.6)	93.5 (92.5, 94.5)	94.0 (93.4, 94.7)	−0.5 (−1.7, 0.7)
	55–74 years	84.0 (83.1, 84.8)	84.8 (84.2, 85.4)	−0.9 (−1.9, 0.2)	80.2 (79.2, 81.2)	80.8 (80.0, 81.5)	−0.6 (−1.8, 0.7)	86.8 (83.1, 90.5)	86.3 (83.3, 89.2)	0.6 (−4.2, 5.3)	87.3 (86.5, 88.2)	88.0 (87.4, 88.6)	−0.6 (−1.7, 0.4)	87.4 (86.7, 88.1)	88.7 (88.3, 89.2)	−1.3 (−2.1, −0.5)
	75+ years	74.9 (73.5, 76.3)	75.9 (74.8, 76.9)	−0.9 (−2.7, 0.8)	72.5 (70.9, 74.1)	74.2 (73.1, 75.3)	−1.7 (−3.6, 0.2)	72.6 (65.8, 79.4)	71.6 (66.2, 77.1)	1.0 (−7.7, 9.7)	78.9 (77.4, 80.5)	80.5 (79.4, 81.5)	−1.5 (−3.4, 0.3)	79.8 (78.7, 81.0)	81.6 (80.8, 82.3)	−1.8 (−3.1, −0.4)

*Note*: Model‐based estimate of relative survival, age‐standardized using the sex‐specific age distributions of patients diagnosed between March 2020 and December 2020. The expected relative survival (No pandemic) was based on patients diagnosed in 2011–2019, with extrapolated log‐linear time trend of excess mortality.

In Denmark, the 1‐year RS during the pandemic was 84.5% in women, which was 1.2 (0.4, 1.9) pp lower than expected, and 82.1% in men, which was 0.9 (0.1, 1.7) pp lower than expected. These numbers correspond to 226 excess deaths in women and 184 in men. In Finland, the 1‐year RS during the pandemic was 82.2% in women, which was 1.0 (0.1, 1.8) pp lower than expected, and 78.9% in men, which was 1.1 (0.2, 2.1) pp lower than expected. These numbers correspond to 150 excess deaths in women and 192 in men. In Norway, the 1‐year RS was 85.5% in women, which was 1.2 (0.3, 2.0) pp lower than expected, and 85.6% in men, which was 0.9 (0.1, 1.7) pp lower than expected. These numbers correspond to 185 excess deaths in women and 164 in men. In Sweden, the 1‐year RS was 85.3% in women, which was 0.5 (−0.2, 1.2) pp lower than expected, and 85.8% in men, which was 1.4 (0.7, 2.0) pp lower than expected. The result for Swedish women was not statistically significant. These results correspond to 126 excess deaths in women and 388 in men. In Iceland, there was no significant decrease in 1‐year RS. Significant increases were only found in Icelandic women, where the 1‐year RS was 86.0%, which was 4.3 (0.6, 8.0) pp higher than expected. In summary, the number of excess deaths was 410 for Denmark, 342 for Finland, 349 for Norway, and 514 for Sweden.

Excess mortality of patients with cancer increased significantly in men in every Nordic country except Iceland, and in women in all Nordic countries except Iceland and Sweden (Table 3). EMRs ranged from 1.06 to 1.12, with the highest EMR of 1.12 observed in Swedish men. The highest age‐specific EMR of 1.31 was observed in Finnish men aged 0–54 years.

**TABLE 3:** One‐year excess mortality ratio (EMR) of patients diagnosed with cancer between March 2020 and December 2020 in Denmark, Finland, Iceland, Norway, and Sweden.

		Denmark	Finland	Iceland	Norway	Sweden
		EMR (95% CI)	EMR (95% CI)	EMR (95% CI)	EMR (95% CI)	EMR (95% CI)
Women	All ages	1.09 (1.03, 1.16)	1.07 (1.01, 1.14)	0.74 (0.56, 0.97)	1.10 (1.03, 1.18)	1.04 (0.99, 1.10)
	0–54 years	1.09 (0.87, 1.35)	0.87 (0.66, 1.15)	1.34 (0.53, 3.40)	1.11 (0.86, 1.42)	1.00 (0.81, 1.22)
	55–74 years	1.13 (1.04, 1.23)	1.03 (0.94, 1.12)	0.59 (0.38, 0.92)	1.11 (1.00, 1.23)	1.00 (0.92, 1.07)
	75+ years	1.06 (0.97, 1.15)	1.12 (1.04, 1.22)	0.79 (0.55, 1.16)	1.10 (1.00, 1.21)	1.09 (1.02, 1.17)
Men	All ages	1.06 (1.00, 1.11)	1.07 (1.01, 1.12)	0.96 (0.74, 1.24)	1.08 (1.02, 1.15)	1.12 (1.06, 1.17)
	0–54 years	1.14 (0.93, 1.39)	1.31 (1.05, 1.63)	0.92 (0.32, 2.66)	1.15 (0.92, 1.45)	1.09 (0.90, 1.33)
	55–74 years	1.06 (0.99, 1.14)	1.03 (0.97, 1.11)	0.97 (0.67, 1.42)	1.07 (0.98, 1.16)	1.13 (1.05, 1.21)
	75+ years	1.04 (0.96, 1.13)	1.08 (0.99, 1.18)	0.95 (0.66, 1.38)	1.09 (0.99, 1.21)	1.11 (1.03, 1.20)

*Note*: Excess mortality ratio (EMR) is defined as the ratio of 1‐year excess mortality in patients diagnosed with cancer  between March 2020 and December 2020 and their expected excess mortality that was based on patients diagnosed in 2011–2019, with extrapolated log‐linear time trend of excess mortality.

In Denmark, the EMR was 1.09 (1.03, 1.16) in women and 1.06 (1.00, 1.11) in men; in Danish women aged 55–74 years, the EMR was 1.13 (1.04, 1.23). In Finland, the EMR was 1.07 (1.01, 1.14) in women and 1.07 (1.01, 1.12) in men; in Finnish men aged 0–54 years, the EMR was 1.31 (1.05, 1.63); and in Finnish women aged 75 years and older, the EMR was 1.12 (1.04, 1.22). In Norway, the EMR was 1.10 (1.03, 1.18) in women and 1.08 (1.02, 1.15) in men; in Norwegian women aged 55–74 years, the EMR was 1.11 (1.00, 1.23). In Sweden, the EMR was 1.12 (1.06, 1.17) in men; in Swedish women aged 75 years and older, the EMR was 1.09 (1.02, 1.17); in Swedish men aged 55–74 years, the EMR was 1.13 (1.05, 1.21); and in Swedish men aged 75 years and older, the EMR was 1.11 (1.03, 1.20). Significant decreases in excess mortality of patients were found in Icelandic women, with the EMR being 0.74 (0.56, 0.97); in Icelandic women aged 55–74 years, the EMR was 0.59 (0.38, 0.92).

Results of 1‐year RS for specific cancer sites are shown in Table 4 and EMRs in Table S2. There were no significant decreases in 1‐year RS or increases in EMRs for breast or prostate cancer in any of the four largest Nordic countries (Denmark, Finland, Norway, or Sweden). In colorectal cancer, however, 1‐year RS decreased by 3.4 (1.2, 5.6) pp in Danish women, 3.6 (1.7, 5.5) pp in Swedish women, and 3.2 (1.1, 5.4) pp in Norwegian men. These correspond to EMRs of 1.27 (1.08, 1.48), 1.27 (1.12, 1.43), and 1.28 (1.09, 1.49), respectively. In lung cancer, 1‐year RS decreased by 2.6 (0.0, 5.2) pp in Danish women, corresponding to an EMR of 1.12 (1.03, 1.22). In pancreatic cancer, 1‐year RS decreased by 5.6 (1.2, 10.0) pp in Finnish women, corresponding to an EMR of 1.20 (1.06, 1.37). In hematopoietic cancer, 1‐year RS decreased by 3.9 (1.2, 6.6) pp in Finnish women and by 2.2 (0.2, 4.2) pp in Swedish women. These correspond to EMRs of 1.29 (1.08, 1.54) and 1.22 (1.03, 1.44), respectively. In liver cancer, 1‐year RS decreased by 10.2 (3.3, 17.1) pp in Finnish men and by 7.2 (1.3, 13.1) pp in Swedish men. These correspond to EMRs of 1.30 (1.08, 1.57) and 1.23 (1.04, 1.46), respectively. Significant increases in 1‐year RS were only found in Norwegian patients with prostate cancer and Swedish patients with breast cancer, with increases of 1.3 (0.9, 1.7) pp and 0.6 (0.0, 1.1) pp, respectively.

**TABLE 4:** One‐year relative survival of patients with cancer during the pandemic (Pandemic), the expected survival without the pandemic (No pandemic), and their difference (Difference) for all sites and for breast, colorectal, lung, pancreatic, hematopoietic, and liver cancer in Denmark, Finland, Norway, and Sweden.

		Denmark			Finland			Norway			Sweden		
		Pandemic	No pandemic	Difference	Pandemic	No pandemic	Difference	Pandemic	No pandemic	Difference	Pandemic	No pandemic	Difference
Women	All sites	84.5 (83.9, 85.2)	85.7 (85.3, 86.1)	−1.2 (−1.9, −0.4)	82.2 (81.5, 82.9)	83.1 (82.6, 83.7)	−1.0 (−1.8, −0.1)	85.5 (84.8, 86.2)	86.6 (86.2, 87.1)	−1.2 (−2.0, −0.3)	85.3 (84.8, 85.9)	85.8 (85.4, 86.2)	−0.5 (−1.2, 0.2)
	Breast	97.2 (96.6, 97.8)	97.3 (96.9, 97.8)	−0.1 (−0.8, 0.7)	96.9 (96.3, 97.6)	97.3 (96.9, 97.8)	−0.4 (−1.2, 0.4)	98.1 (97.4, 98.7)	97.5 (97.0, 98.0)	0.5 (−0.3, 1.3)	98.6 (98.2, 99.0)	98.1 (97.7, 98.4)	0.6 (0.0, 1.1)
	Colorectal	83.4 (81.6, 85.3)	86.9 (85.7, 88.0)	−3.4 (−5.6, −1.2)	83.6 (81.5, 85.6)	84.4 (82.9, 85.9)	−0.8 (−3.4, 1.7)	84.6 (82.9, 86.4)	85.8 (84.5, 87.1)	−1.2 (−3.4, 1.0)	81.5 (80.0, 83.1)	85.1 (84.0, 86.2)	−3.6 (−5.5, −1.7)
	Lung	59.2 (57.1, 61.3)	61.8 (60.3, 63.3)	−2.6 (−5.2, 0.0)	47.5 (44.4, 50.7)	47.5 (45.0, 49.9)	0.1 (−4.0, 4.1)	59.7 (57.0, 62.4)	60.6 (58.6, 62.6)	−0.9 (−4.2, 2.5)	58.3 (56.0, 60.5)	59.1 (57.4, 60.7)	−0.8 (−3.6, 2.0)
	Pancreas	34.4 (30.0, 38.7)	35.8 (32.6, 39.0)	−1.5 (−6.8, 3.9)	27.2 (23.7, 30.7)	32.8 (30.1, 35.5)	−5.6 (−10.0, −1.2)	37.3 (31.9, 42.7)	38.0 (34.0, 42.1)	−0.8 (−7.5, 6.0)	38.1 (34.5, 41.6)	37.7 (34.9, 40.6)	0.4 (−4.2, 4.9)
	Hematopoietic	89.1 (87.4, 90.8)	90.9 (89.8, 92.0)	−1.9 (−3.9, 0.2)	80.9 (78.6, 83.2)	84.8 (83.4, 86.3)	−3.9 (−6.6, −1.2)	86.8 (84.7, 88.8)	88.7 (87.3, 90.1)	−1.9 (−4.4, 0.6)	86.1 (84.5, 87.8)	88.3 (87.3, 89.4)	−2.2 (−4.2, −0.2)
	Liver	39.1 (31.0, 47.3)	40.7 (34.3, 47.0)	−1.5 (−11.8, 8.7)	37.8 (29.7, 45.8)	44.1 (38.4, 49.9)	−6.4 (−16.2, 3.5)	48.9 (38.8, 59.0)	50.9 (42.9, 58.8)	−2.0 (−14.8, 10.8)	47.0 (39.7, 54.3)	51.0 (46.1, 55.9)	−4.0 (−12.8, 4.8)
Men	All sites	82.1 (81.5, 82.8)	83.0 (82.6, 83.5)	−0.9 (−1.7, −0.1)	78.9 (78.1, 79.6)	80.0 (79.4, 80.5)	−1.1 (−2.1, −0.2)	85.6 (85.0, 86.3)	86.5 (86.0, 87.0)	−0.9 (−1.7, −0.1)	85.8 (85.3, 86.3)	87.1 (86.8, 87.5)	−1.4 (−2.0, −0.7)
	Prostate	97.4 (96.7, 98.1)	97.1 (96.6, 97.7)	0.3 (−0.6, 1.2)	97.7 (97.0, 98.4)	97.8 (97.3, 98.3)	−0.1 (−0.9, 0.7)	100.0 (NA, NA)	98.7 (98.3, 99.1)	1.3 (0.9, 1.7)	98.5 (98.1, 98.9)	98.8 (98.5, 99.0)	−0.3 (−0.8, 0.2)
	Colorectal	85.7 (84.0, 87.4)	87.3 (86.2, 88.4)	−1.6 (−3.7, 0.4)	82.6 (80.6, 84.5)	83.0 (81.5, 84.5)	−0.4 (−2.9, 2.0)	84.0 (82.2, 85.7)	87.2 (86.0, 88.4)	−3.2 (−5.4, −1.1)	85.7 (84.3, 87.1)	85.7 (84.6, 86.7)	0.0 (−1.7, 1.8)
	Lung	51.3 (49.1, 53.5)	53.3 (51.6, 54.9)	−2.0 (−4.7, 0.8)	39.1 (36.5, 41.7)	40.9 (39.0, 42.8)	−1.8 (−5.0, 1.4)	52.6 (49.9, 55.3)	53.1 (51.1, 55.1)	−0.5 (−3.8, 2.9)	51.3 (48.7, 53.8)	51.4 (49.6, 53.1)	−0.1 (−3.2, 3.0)
	Pancreas	36.5 (32.2, 40.8)	37.0 (33.8, 40.1)	−0.4 (−5.8, 4.9)	25.9 (22.4, 29.4)	28.1 (25.4, 30.8)	−2.2 (−6.6, 2.2)	37.4 (32.4, 42.4)	38.8 (34.9, 42.6)	−1.3 (−7.6, 5.0)	36.9 (33.2, 40.6)	39.8 (37.0, 42.6)	−2.9 (−7.5, 1.7)
	Hematopoietic	87.3 (85.6, 88.9)	87.0 (85.8, 88.2)	0.2 (−1.8, 2.2)	81.2 (79.2, 83.3)	83.1 (81.6, 84.5)	−1.8 (−4.3, 0.7)	87.3 (85.5, 89.1)	87.6 (86.3, 88.9)	−0.3 (−2.5, 1.9)	87.0 (85.6, 88.5)	87.1 (86.1, 88.1)	−0.1 (−1.9, 1.7)
	Liver	47.1 (40.9, 53.3)	47.8 (43.6, 52.0)	−0.7 (−8.1, 6.8)	35.1 (29.5, 40.7)	45.3 (41.1, 49.4)	−10.2 (−17.1, −3.3)	52.2 (44.7, 59.7)	47.8 (41.5, 54.2)	4.4 (−5.4, 14.2)	48.5 (43.7, 53.3)	55.7 (52.3, 59.1)	−7.2 (−13.1, −1.3)

*Note*: Model‐based estimate of relative survival, age‐standardized using the sex‐specific age distributions of patients diagnosed between March 2020 and December 2020. The expected relative survival (No pandemic) was based on patients diagnosed in 2011–2019, with extrapolated log‐linear time trend of excess mortality.

## 4 DISCUSSION

During the first year of the COVID‐19 pandemic, we observed consistent reductions in 1‐year RS in patients with cancer across all Nordic countries except Iceland, with corresponding increases in excess mortality. Particularly, men and women aged 75 years and older in Sweden, and women aged 75 years and older in Finland, experienced reductions in 1‐year RS of approximately 2–3 percentage points. The largest increases in excess mortality were seen in Swedish men (12%) and Norwegian women (10%). Notably, the largest site‐specific reductions in 1‐year RS, exceeding 7 percentage points, were observed in men with liver cancer in Finland and Sweden.

Before the COVID‐19 pandemic, age‐standardized cancer mortality had steadily declined and 1‐year relative survival had improved across the Nordic countries.^5^ Our findings align with a growing body of evidence indicating that the pandemic negatively affected short‐term cancer survival. Population‐based studies from Belgium, Canada, Northern Ireland, and the UK reported lower 1‐year RS or overall survival for various cancer sites in 2020 compared to previous years.^8^, ^9^, ^10^, ^11^ In contrast, population‐level cancer mortality has shown continued declines even during the pandemic. Age‐standardized cancer mortality continued to decline in the Nordic countries through 2020, and in the United States, the death rate with cancer as the underlying cause was lower in 2020 than in 2019.^5^, ^23^ However, these figures reflect mortality in patients diagnosed years earlier and rely on cause‐of‐death information, which may mask short‐term effects among newly diagnosed patients. Additionally, modeling studies have predicted future increases in cancer mortality related to diagnostic and treatment delays during the pandemic.^24^, ^25^

The COVID‐19 pandemic may have disproportionately affected patients with cancer, leading to reduced survival among those diagnosed or treated during the pandemic. Excess mortality may have been driven by multiple factors, including reluctance to seek medical care, reduced diagnostic activity, and disruptions in cancer screening programs.^2^ Patients with cancer may have been diagnosed at more advanced stages, possibly resulting in poorer treatment outcomes. Delays and modifications in treatment might have also affected outcomes, whereas immunocompromised patients faced additional risks from COVID‐19 infections.^26^, ^27^ Those who had completed active treatment may have experienced inadequate surveillance, resulting in delayed detection and treatment of recurrences.^27^ Furthermore, patients with cancer infected with COVID‐19 had higher mortality rates, exacerbating the overall impact.^28^

Overall, the Nordic countries maintained access to healthcare services for non‐COVID‐19 conditions, although fewer patients sought care in the early months of the pandemic. Our previous research shows substantial and consistent declines in new cancer diagnoses in Denmark, Finland, Norway, and Sweden during the first year of the pandemic, particularly during the first few months.^2^ Sweden, which experienced the highest COVID‐19 incidence and mortality in 2020, also had the largest decline in cancer survival among men. In contrast, Iceland, with the lowest pandemic burden and a smaller, more contained healthcare system combined with geographical isolation, showed no significant decrease in RS. Screening continuity also varied: Denmark and Finland largely maintained screening programs, albeit with lower participation, while temporary halts occurred in Sweden, Norway, and Iceland. The timing and intensity of restrictions, public compliance, and reduced diagnostic activity all likely contributed to the heterogeneous outcomes observed. Cancer care services in the Nordic countries remained largely operational throughout the pandemic.^2^ Instead, the observed effects may be more attributable to changes in individual health‐seeking behavior, such as delayed presentation or reduced engagement with healthcare services. Our estimates of the pandemic's impact on cancer outcomes reflect the combined effects of several factors such as delayed diagnoses, shifts in the types of cancer cases diagnosed, altered treatment pathways, changes in individual healthcare behavior, and COVID‐19‐related risks. Disentangling these factors was beyond the scope of this study.

Excess mortality ratios were generally higher and 1‐year relative survival lower among older patients with cancer. Notably, men and women aged 75 years and older in Sweden, and women aged 75 years and older in Finland, experienced approximately 2–3 percentage points reductions in RS. Several factors might explain this age‐related disparity. Older individuals are more likely to be frail and have comorbidities that may limit treatment options, which may have made them more susceptible to both the direct and indirect impacts of the pandemic.^29^ Healthcare providers may have modified or postponed treatments in older patients due to concerns about immune suppression or treatment‐related risks during a period of heightened COVID‐19 transmission. In addition, older patients may have delayed seeking care due to fear of infection or faced greater barriers to accessing healthcare services during lockdowns and healthcare system strain. Together, these factors likely contributed to worsened cancer outcomes in the older age groups during the pandemic.

The variation in site‐specific 1‐year RS highlights the differential impact of the pandemic across cancer types. Notably, breast and prostate cancers, which are typically characterized by high survival rates and often detected through screening, did not show significant reductions in RS. This may reflect relatively small disruptions in care for these cancers, as well as the less aggressive nature and slower progression of early‐stage cases. In contrast, more pronounced declines in RS were observed for liver, pancreatic, colorectal, hematopoietic, and lung cancers. These cancers often present symptomatically and can progress rapidly, making timely diagnosis and intervention critical. Lung, liver, and pancreatic cancers are among the most lethal malignancies, and even minor delays in diagnosis or treatment might worsen prognosis. Hematopoietic cancers, which include leukemias and lymphomas, also showed notable decreases. Many patients with hematopoietic cancer undergo immunosuppressive therapies that increase the risk of severe COVID‐19 infection. Patients with lung cancer, especially those with impaired pulmonary function, may have experienced worse outcomes due to both diagnostic delays and the direct respiratory effects of SARS‐CoV‐2. These findings suggest that the pandemic may have worsened the prognosis of cancers that progress rapidly, involve immunosuppressive treatments, or result in greater sensitivity to respiratory complications.

Population‐based cancer survival estimates during the COVID‐19 pandemic require careful interpretation.^27^ Cancer survival depends on many factors that the pandemic may have confounded through case composition and changes in population mortality.^2^, ^4^, ^27^, ^30^ Screening programs were suspended, fewer patients presented with mild or non‐specific symptoms, and healthcare resources were shifted.^1^, ^2^, ^3^ This likely contributed to a lower proportion of screen‐detected or low‐risk cancers during the pandemic.^30^ Disproportionate decreases in early‐stage cancers diagnosed outside of screening programs may have skewed the diagnosed cases toward those with more advanced disease. Since low‐risk cases can inflate survival statistics, their decrease during the pandemic could make survival appear worse than it is. Relative survival methods also use general population life tables to estimate non‐cancer mortality. If patients with cancer experienced different non‐cancer mortality during the pandemic, this may have affected relative survival estimates.^27^

To our knowledge, this is the first study to investigate the impact of the COVID‐19 pandemic on relative survival and excess mortality in a large population‐based cohort of patients with cancer, including follow‐up of over 27 million people in the Nordic countries. The use of high‐quality data from national cancer registries and official death statistics provides robustness to the results. Similar data collection and cancer coding methods across the Nordic cancer registries increase the consistency and comparability of the results.

This study has some limitations. Tumor stage was not used due to incomplete recording across the Nordic cancer registries.^15^ This prevents us from assessing whether the observed reduction in relative survival was due to differences in the stage distribution of the diagnosed cases. In addition, follow‐up ended at 1 year after diagnosis, which limits our ability to assess the long‐term impact of the pandemic on cancer survival. We acknowledge that nonlinearities in survival trends may have affected our estimates. However, the assumption of a log‐linear trend was considered appropriate as the observed changes in survival have been stable in many cancer types during the last decades.

Across Denmark, Finland, Norway, and Sweden, 1‐year relative survival declined, and excess mortality increased among patients diagnosed with cancer during the COVID‐19 pandemic. These changes were generally larger in older patients and in those with more aggressive cancers. However, further research is needed to determine causality and generalizability of the results. Overall, our findings highlight the need for prepared and resilient healthcare systems that can maintain adequate cancer services even during pandemics and public health emergencies. Developing strategies to ensure continuous healthcare access and address reluctance to seek healthcare is essential for improving cancer outcomes in such circumstances. Future research should elucidate the mechanisms by which the pandemic disrupted healthcare systems and the delivery of cancer care and assess the long‐term impacts on patients with cancer and other chronic diseases.

## AUTHOR CONTRIBUTIONS

**Fernando Gonzalez Yli‐Mäyry:** Writing – original draft; visualization; writing – review and editing. **Tomas Tanskanen:** Conceptualization; writing – review and editing; formal analysis; data curation; methodology; writing – original draft. **Karri Seppä:** Conceptualization; writing – review and editing. **Anna L. V. Johansson:** Conceptualization; writing – review and editing; formal analysis; data curation. **Charlotte Wessel Skovlund:** Formal analysis; data curation; writing – review and editing. **Lina Steinrud Mørch:** Conceptualization; writing – review and editing. **Søren Friis:** Writing – review and editing. **Simon Mathis Kønig:** Writing – review and editing; formal analysis; data curation. **Tom Børge Johannesen:** Writing – review and editing; formal analysis; data curation. **Tor Åge Myklebust:** Writing – review and editing; formal analysis; data curation. **Sasha Pejicic:** Writing – review and editing; formal analysis; data curation. **David Pettersson:** Writing – review and editing; formal analysis; data curation. **Eva María Guðmundsdóttir:** Formal analysis; writing – review and editing; data curation. **Sirpa Heinävaara:** Writing – review and editing. **Nea Malila:** Writing – review and editing. **Joonas Miettinen:** Writing – review and editing; formal analysis; data curation. **Johan Ahlgren:** Writing – review and editing. **Giske Ursin:** Conceptualization; writing – review and editing; data curation; formal analysis. **Janne Pitkäniemi:** Writing – review and editing; conceptualization.

## CONFLICT OF INTEREST STATEMENT

The authors declare no conflicts of interest.

## Supporting information


**Table S1.** Annual numbers of cancer deaths, all deaths, and incident cancers in men and women in the Nordic countries between 2011 and 2021.
**Table S2**. 1‐year excess mortality ratio (EMR) of patients diagnosed with cancer during the pandemic with respect to a hypothetical scenario without the pandemic for all sites and for breast, colorectal, hematopoietic, liver, lung, pancreatic, and prostate cancer in Denmark, Finland, Norway and, Sweden.

## Data Availability

This study was based on national health registry data. The data are available from the registry holder of each specific health registry under the use of appropriate ethical and legal permissions, including the GDPR. Further details and other data that support the findings of this study are available from the corresponding author upon request.
