# Supplementary material for: Changes in 1‐year relative survival of patients with cancer during the COVID‐19 pandemic in Denmark, Finland, Iceland, Norway, and Sweden: A population‐based cohort study
Source: Int J Cancer. 2026 Jan 21;158(12):3121–31. doi: 10.1002/ijc.70336 (PMC13106915; doi:10.1002/ijc.70336)
Supplement: Supplementary file 1 — Table S1. Annual numbers of cancer deaths, all deaths, and incident cancers in men and women in the Nordic countries between 2011 and 2021. Table S2. 1‐year excess mortality ratio (EMR) of patients diagnosed with cancer during the pandemic with respect to a hypothetical scenario without the pandemic for all sites and for breast, colorectal, hematopoietic, liver, lung, pancreatic, and prostate cancer in Denmark, Finland, Norway and, Sweden. [file IJC-158-3121-s001.pdf]

Changes in 1-year relative survival of patients with cancer during the COVID-19 pandemic in Denmark, Finland, Iceland, Norway, and Sweden: A population-based cohort study

Authors

Fernando Gonzalez Yli-Mäyry, Tomas Tanskanen, Karri Seppä, Anna L V Johansson, Charlotte Wessel Skovlund, Lina Steinrud Mørch, Søren Friis, Simon Mathis Kønig, Tom Børge Johannesen, Tor Åge Myklebust, Sasha Pejicic, David Pettersson, Eva María Guðmundsdóttir, Sirpa Heinävaara, Nea Malila, Joonas Miettinen, Johan Ahlgren, Giske Ursin, Janne Pitkäniemi

Table of contents

- Supplementary Table 1
- Supplementary Table 2

Supplementary Table 1. Annual numbers of cancer deaths, all deaths, and incident cancers in men and women in the Nordic countries between 2011 and 2021.

Sex	Period	All countries			DENMARK			FINLAND			ICELAND			NORWAY			SWEDEN		
		Cancer deaths	All deaths	Incident cancers	Cancer deaths	All deaths	Incident cancers	Cancer deaths	All deaths	Incident cancers	Cancer deaths	All deaths	Incident cancers	Cancer deaths	All deaths	Incident cancers	Cancer deaths	All deaths	Incident cancers
Women	2011	26515	120498	69105	7023	26577	17760	5591	25236	14166	392	1044	751	4614	21307	13091	8895	46334	23337
	2012–2018[1]	+153	-62	+1254	+1	-10	+236	+54	+204	+284	+8	+13	+9	+36	-78	+318	+55	-191	+406
	2019	27742	119985	79141	7029	26504	19651	6024	26869	16441	457	1120	824	4898	20685	15636	9334	44807	26589
	2020	27513	124664	76445	6940	26651	19112	6091	27502	15737	449	1141	852	4883	20554	15511	9150	48816	25233
	2021	27546	123358	79987	6899	27991	19707	6048	28532	16269	465	1206	898	5088	21382	16427	9046	45453	26686
Men	2011	30002	115964	76181	7823	25939	18475	6139	25365	14791	455	1038	732	5485	20067	15436	10100	43555	26747
	2012–2018	+198	+479	+1520	+39	+217	+311	+103	+214	+344	+5	+23	+14	+20	-16	+291	+31	+69	+560
	2019	31586	119737	88342	8134	27455	20961	6962	27077	17542	497	1159	843	5647	19938	17765	10346	44108	31231
	2020	31615	126613	85234	8078	27995	20552	6997	27931	17283	512	1167	846	5744	20024	18072	10284	49496	28481
	2021	31251	125205	88497	8082	29188	21429	7204	29170	17686	512	1253	910	5680	20520	18370	9773	46327	30102
All	2011	56517	236462	145286	14846	52516	36235	11730	50601	28957	847	2082	1483	10099	41374	28527	18995	89889	50084
	2012–2018[1]	+351	+418	+2775	+40	+206	+547	+157	+418	+628	+13	+35	+23	+56	-94	+609	+86	-122	+967
	2019	59328	239722	167483	15163	53959	40612	12986	53946	33983	954	2279	1667	10545	40623	33401	19680	88915	57820
	2020	59128	251277	161679	15018	54646	39664	13088	55433	33020	961	2308	1698	10627	40578	33583	19434	98312	53714
	2021	58797	248563	168484	14981	57179	41136	13252	57702	33955	977	2459	1808	10768	41902	34797	18819	91780	56788

[1]Average of the differences between consecutive calendar years in the numbers of cancer deaths, all deaths, and incident cancers.

Supplementary Table 2. 1-year excess mortality ratio[1] (EMR) of patients diagnosed with cancer during the pandemic with respect to a hypothetical scenario without the pandemic for all sites and for breast, colorectal, hematopoietic, liver, lung, pancreatic, and prostate cancer in Denmark, Finland, Norway, and Sweden.

		Denmark	Finland	Norway	Sweden
		**EMR (95% CI)**	**EMR (95% CI)**	**EMR (95% CI)**	**EMR (95% CI)**
**Women**	**All sites**	1.09 (1.03, 1.16)	1.07 (1.01, 1.14)	1.10 (1.03, 1.18)	1.04 (0.99, 1.10)
	**Breast**	1.05 (0.80, 1.38)	1.18 (0.91, 1.54)	0.80 (0.55, 1.17)	0.71 (0.51, 1.00)
	**Colorectal**	1.27 (1.08, 1.48)	1.08 (0.91, 1.29)	1.08 (0.92, 1.27)	1.27 (1.12, 1.43)
	**Lung**	1.12 (1.03, 1.22)	1.01 (0.90, 1.13)	1.04 (0.93, 1.17)	1.05 (0.96, 1.15)
	**Pancreas**	1.06 (0.91, 1.23)	1.20 (1.06, 1.37)	1.05 (0.87, 1.27)	1.00 (0.88, 1.13)
	**Hematopoietic**	1.23 (0.99, 1.52)	1.29 (1.08, 1.54)	1.17 (0.94, 1.46)	1.22 (1.03, 1.44)
	**Liver**	1.06 (0.79, 1.41)	1.21 (0.92, 1.59)	1.06 (0.72, 1.56)	1.21 (0.95, 1.55)
**Men**	**All sites**	1.06 (1.00, 1.11)	1.07 (1.01, 1.12)	1.08 (1.02, 1.15)	1.12 (1.06, 1.17)
	**Prostate**	0.90 (0.64, 1.27)	1.04 (0.72, 1.50)	NA (NA, NA)	1.23 (0.85, 1.77)
	**Colorectal**	1.17 (0.99, 1.37)	1.05 (0.89, 1.23)	1.28 (1.09, 1.49)	1.01 (0.88, 1.15)
	**Lung**	1.08 (1.00, 1.17)	1.08 (0.99, 1.18)	1.04 (0.94, 1.15)	1.03 (0.94, 1.12)
	**Pancreas**	1.02 (0.88, 1.19)	1.06 (0.94, 1.21)	1.07 (0.90, 1.27)	1.10 (0.97, 1.25)
	**Hematopoietic**	0.97 (0.81, 1.15)	1.13 (0.96, 1.33)	1.04 (0.86, 1.26)	1.02 (0.87, 1.18)
	**Liver**	1.02 (0.82, 1.25)	1.30 (1.08, 1.57)	0.90 (0.68, 1.19)	1.23 (1.04, 1.46)

[1]Excess mortality ratio (EMR) is defined as the ratio of 1-year excess mortality in patients diagnosed with cancer between March 2020 and December 2020 and their expected excess mortality that was based on patients diagnosed in 2011–2019 with extrapolated log-linear time trend of excess mortality.
